# Antibodies to the DNA-directed RNA polymerase II subunit RPB1 occur with highest frequency in centenarians

**DOI:** 10.1186/s12979-016-0064-1

**Published:** 2016-03-22

**Authors:** Jungwon Han, So-Hyun Park, Dong-Jo Kim, Hyori Kim, Yoon-Ho Choi, Jong-Hyuk Lee, Sang Chul Park, Junho Chung

**Affiliations:** Department of Biochemistry and Molecular Biology, Seoul National University College of Medicine, Seoul, 00380 South Korea; Department of Biomedical Science, Seoul National University College of Medicine, Seoul, 00380 South Korea; Cancer Research Institute, Seoul National University College of Medicine, Seoul, 00380 South Korea; Department of Medicine, Center for Health Promotion, Samsung Medical Center, Sungkyunkwan University School of Medicine, Seoul, 06351 South Korea; Present Address: Seoul Animal Medical Center, Gyenggi-do, 12790 South Korea; Present Address: Celltrion, Incheon, 22014 South Korea; Present Address: Daegu Gyeongbuk Institute of Science and Technology, Deagu, 42988 South Korea

**Keywords:** Centenarians, Antibody, Phage display, RPB1, CTD

## Abstract

**Background:**

Recently, phage display technology has made it possible to define the circulating repertoire of humoral immunity. This study was designed to define the circulating antibodies specific to centenarians.

**Results:**

We used a phage-displayed combinatorial peptide library to screen for peptides (YSATLRY and YSPTLFY) that preferentially react with the IgG fraction of centenarians aged 100–105 years. Centenarian sera binds to YSATLRY and YSPTLFY with higher frequency than that of healthy volunteers aged 60–79 years or healthy volunteers younger than or equal to 43 years of age. We prepared polyclonal antibodies to YSATLRY from human sera to immunoprecipitate the native antigen, which was identified as the carboxy-terminal domain (CTD) of DNA-directed RNA polymerase II subunit RPB1. The RBP1 CTD contains multiple YSPTSPS repeats, which are significantly homologous to YSATLRY and YSPTLFY. The immunoprecipitated RPB1 had significantly slower mobility than did RPB1 in cell lysates, and the polyclonal antibodies reacted with CTD peptide, depending on the phosphorylation pattern. Therefore, it appears that the polyclonal antibodies preferentially bind to highly phosphorylated RPB1. We also confirmed that human monoclonal antibodies reactive to both YSATLRY and YSPTLFY bound to the phosphorylated YSPTSPS motif.

**Conclusions:**

This study showed that centenarians possess IgG antibodies that are reactive to YSATLRY and YSPTLFY, mimicking the phosphorylated form of the YSPTSPS motif (CTD of RPB1), at a much higher frequency than that of the average population.

**Electronic supplementary material:**

The online version of this article (doi:10.1186/s12979-016-0064-1) contains supplementary material, which is available to authorized users.

## Findings

Humoral immunity has evolved to protect the host. For an individual, it is one of the greatest biological advantages to harbor an effective repertoire of humoral immunity against hostile agents like bacteria, viruses, or cancers [1]. There has been a long-standing question whether centenarians possess a special humoral immunity repertoire that enables longer survival than that of the general population. A phage-displayed combinatorial peptide library made it possible to enrich for antibody-reactive peptides [2]. These peptide sequences can be used to identify specific antigens. In this study, we used a phage-displayed combinatorial peptide library to screen for peptides that preferentially react to the IgG fraction of centenarians and identified the antigen mimicked by these peptides.

**Fig. 1:**
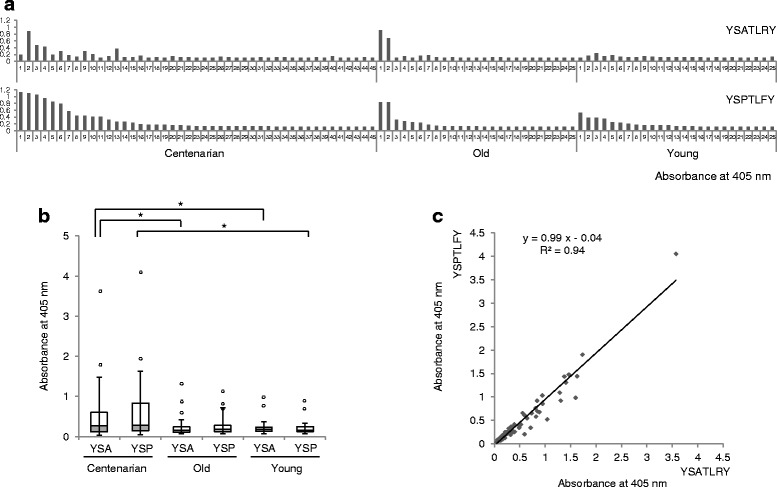
Defining the humoral repertoire of centenarians with peptide mimotopes. **a** Microtiter plates were coated with anti-human IgG antibodies. After blocking, sera from individual centenarians (Centenarian group), healthy volunteers aged 60–79 years (Old group), and healthy volunteers younger than or equal to 43 years (Young group) were added to the wells. Phage-displaying peptide YSATLRY or YSPTLFY was added, and the amount of bound phage was determined using the anti-M13 antibody HRP conjugate and ABTS substrate solution. (**b**) Microtiter plates were coated with BSA-conjugated peptides (YSA = YSATLRYGGGSC, YSP = YSPTLFYGGGSC). After blocking, sera from individuals were added to each well. After incubation and washing, HRP-conjugated anti-human IgG (H + L) antibodies and ABTS solution were added sequentially with intermittent washing. * *P* < 0.05. (**c**) R-square calculation from the enzyme immunoassay result shown in B (*R* ^2^ = 0.94)

The sera of three populations were collected, including 45 centenarians aged 100–105 years (defined as the centenarian group), 25 healthy volunteers aged 60–79 years (defined as the old group), and 25 healthy volunteers younger than or equal to 43 years (defined as the young group) (Additional file 1: Table S1). IgG fractions were purified from the centenarian sera by protein G column chromatography, and these fractions were used to enrich phage from the phage-displayed combinatorial peptide library through biopanning (Additional file 2). After the final round of biopanning, phages that were preferentially reactive to the centenarian IgG pool were selected by a phage enzyme immunoassay. Phage clones encoding two highly homologous peptides with YSATLRY and YSPTLFY sequences were strongly enriched in the samples, each of which comprised 20 % of positive clones. These two peptides, either phage-displayed or chemically synthesized and conjugated to bovine serum albumin (BSA), reacted with individual centenarian IgG fractions at a much higher frequency than did IgG fractions of other individuals in the enzyme immunoassays (Fig. 1a and b). Each individual centenarian’s antibody titers to these two peptides were highly correlated; therefore, we hypothesized that these two peptides actually represent the same antigen epitope (Fig. 1c).

To prepare human polyclonal antibodies (pAbs) to YSATLRY, we collected sera from 59 additional healthy volunteers aged 20–40 years and performed enzyme immunoassays to screen for those who had antibodies to the two homologous peptides (Additional file 3: Figure S1). Five volunteers exhibited significant antibody titers to both YSATLRY and YSPTLFY (Additional file 3: Figure S1; volunteers #7, #11, #19, #47, and #50). The pAbs were prepared from these sera using an YSATLRYGGGSC-cross-linked affinity column, and the pAb specificity to the peptides was confirmed by competition enzyme immunoassays (Additional file 4: Figure S2). These results showed that pAb binding to the YSATLRYGGGSC-BSA conjugate coated on microtiter plates was competitively hindered by YSATLRYGGGS in the soluble fraction.

**Fig. 2:**
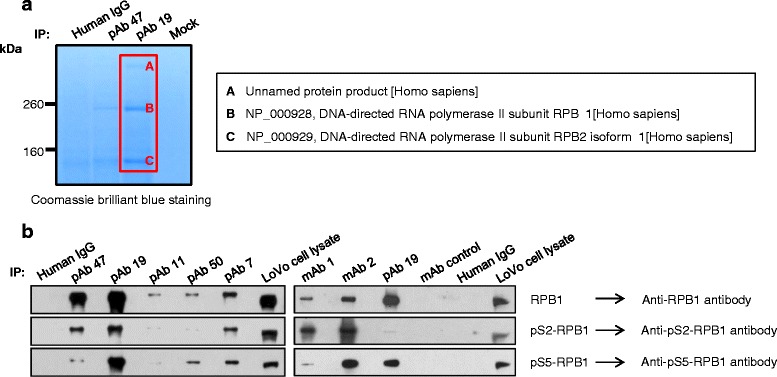
Identification of RPB1 as the antigen recognized by pAb 47, pAb 19, and mAbs. **a** LoVo cell lysates were immunoprecipitated with pAb 47 and pAb 19, and the immunoprecipitates were subjected to 4–12 % Bis-Tris gel electrophoresis. Gels were stained with Coomassie Brilliant Blue, the bands were excised, and their identities were determined using mass spectrometry. (**b**) LoVo cell lysates were immunoprecipitated with pAb or mAb (scFv-human Fc fusion protein). The resulting precipitates were subjected to immunoblot analysis with antibodies specific for RPB1 or phosphorylated RPB1. Normal human pooled IgG fractions and LoVo cell lysates were used as controls

We used the pAbs to identify the antigen by immunoprecipitation analysis and found that many human cell lines contained antigen that was reactive to the pAbs (data not shown). The pAbs prepared from volunteers #47 (pAb 47) and #19 (pAb 19) immunoprecipitated three major protein bands from LoVo cell lysates (Fig. 2a). The proteins were subjected to mass spectrometry analysis, and two of them were identified as DNA-directed RNA polymerase II subunit RPB1 (largest subunit of RNA polymerase II; NP_000928) and DNA-directed RNA polymerase II subunit RPB2 isoform 1 (NP_000929). Both proteins are components of the RNA polymerase II complex [3]; therefore, we concluded that the pAbs immunoprecipitated part of the RNA polymerase II complex. The pAbs reacted with protein that had a similar molecular weight to RPB1 (Fig. 2b). We searched RPB1 amino acid sequences homologous to YSATLRY and YSPTLFY, and found that the RPB1 carboxy-terminal domain (CTD) contains multiple YSPTSPS repeats, which are significantly homologous to YSATLRY and YSPTLFY. The identity of RPB1 was confirmed in immunoprecipitation analysis using the pAbs and immunoblot analysis with anti-RPB1 antibodies (Fig. 2b). Every residue of the YSPTSPS motif can be phosphorylated except for proline [4]; therefore, we tested the phosphorylation status of immunoprecipitated RPB1 CTD using antibodies reactive to YpSPTSPS (pS2-RPB1) and YSPTpSPS (pS5-RPB1). Both antibodies reacted with the immunoprecipitates in the immunoblot, indicating that all immunoprecipitated RPB1 contained both YpSPTSPS and YSPTpSPS motifs (Fig. 2b). Immunoprecipitated RPB1 had significantly slower mobility than did RPB1 in cell lysates, suggesting that the pAbs preferentially bind to highly phosphorylated RPB1 [5, 6]. This preferential binding was confirmed in enzyme immunoassays using conjugates of CTD peptide and BSA (Additional file 5: Figure S3A). pAb 19 did not react with YSPTSPSYSPTSPS (CTD-BSA), YSPTpSPSYSPTSPS (pS5-BSA), or YSPTpSPSYpSPTSPS (pS5S2-BSA) (Additional file 5: Figure S3B). We hypothesized that pAb 19 would bind to the YSPTSPS motif with a different phosphorylation pattern.

To confirm that single antibody molecules reactive to YSATLRY and YSPTLFY also bind to phosphorylated YSPTSPS motifs, we generated monoclonal antibodies (mAbs) from the B-lymphocyte pool of volunteer #19. Briefly, a phage-displayed combinatorial single-chain variable fragment (scFv) library with a complexity of 1.84 × 10^9^ was constructed using mRNA prepared from peripheral mononuclear cell fractions of volunteer #19. Biopanning was performed on YSATLRY, and two clones reactive to both YSATLRY and YSPTLFY were selected. Then, two antibody clones were prepared as scFv-human Fc fusion proteins as described previously [7]. The reactivity of the two mAbs (scFv-human Fc fusion protein) was tested in enzyme immunoassays (Additional file 5: Figure S3B). mAb 2 reacted with YSATLRY-BSA, YSPTLFY-BSA, pS5-BSA, and pS5S2-BSA, but not with nonphosphorylated CTD (CTD-BSA). This result confirmed that YSATLRY and YSPTLFY mimic the phosphorylated CTD motif. The mAb 1 clone did not react with any of the CTD peptides, similar to pAb 19.

The reactivity of mAbs to RPB1 CTD was tested in immunoprecipitation and immunoblot analyses. A band with a molecular weight equivalent to RPB1 was visualized in lanes loaded with mAb 1 and mAb 2 immunoprecipitates (Additional file 6: Figure S4). The identities of these protein bands were confirmed by immunoblot analysis (Fig. 2b). The antibodies that reacted with RPB1 CTD (RPB1), YpSPTSPS (pS2-RPB1), and YSPTpSPS (pS5-RPB1) all reacted with the protein band (Additional file 4: Figure S2B).

In summary, this study showed that centenarians possess IgG antibodies that are reactive to YSATLRY and YSPTLFY, mimicking the phosphorylated form of the YSPTSPS motif (RPB1 CTD) at a much higher frequency than the average population. RNA polymerase has been reported to function as an autoantigen in scleroderma patients [8–11]. It has also been suggested that the repetitive amino acid sequence and high content of charged residues in the RPB1 CTD structure contribute to its role as an autoantigen [12]. However, the life expectancy of patients with scleroderma is not significantly different from healthy individuals [13]. Currently, it is not clear whether autoantibodies provide a protective function to centenarians. To determine whether the subjects possessing IgG antibodies reactive to YSATLRY and YSPTLFY had an overall tendency to produce autoantibodies, we tested antinuclear antibody (ANA) levels in the sera of 45 centenarians and 25 old and 25 young volunteers. Of these, 4 centenarians and 1 old healthy volunteer showed detectable ANA levels. However, there was no correlation between ANA level and the levels of serum antibodies against YSATLRY and YSPTLFY (Additional file 7: Figure S5). The antibodies that recognize phosphorylated CTD of RPB1 might result from the aging process, to which centenarians have been exposed for a longer period than the average population. To our knowledge, this is the first report on autoreactive antibodies against RPB1 CTD in centenarians that occur at a higher frequency than in the average population. The mechanism of how these antibodies are generated and their role in human (patho)physiology are interesting topics that we will pursue in future research.

## Additional files

Additional file 1: Table S1. Age and gender distribution of subjects. (DOCX 18 kb) Additional file 2: Materials and Methods. (DOCX 29 kb) Additional file 3: Figure S1. Enzyme immunoassay to determine antibody titer to YSATLRY (A) and YSPTLFY (B). Wells of a microtiter plate were coated with either YSATLRYGGGSC or YSPTLFYGGGSC peptide conjugated to BSA and blocked with 3 % BSA in PBS. Sera from individuals diluted in 3 % BSA in PBS were added to the wells. After washing with 0.05 % PBST three times, the plates were incubated with HRP-conjugated anti-human IgG antibodies. The washing steps were repeated three times. 2,2′-azino-bis(3-ethylbenzothiazoline-6-sulphonic acid (ABTS) in 0.05 M citric acid buffer (pH 4.0) and 1.0 % H_2_O_2_ were added to each well. OD was measured at 405 nm with a microplate spectrophotometer. Asterisks indicate five selected volunteers for further studies (Volunteer #7 = pAb 7, Volunteer #11 = pAb 11, Volunteer #19 = pAb 19, Volunteer #47 = pAb 47, and Volunteer #50 = pAb 50). (DOCX 150 kb) Additional file 4: Figure S2. Competition enzyme immunoassay using polyclonal antibodies (pAbs) with the peptide YSATLRY. Sera from five volunteers showed reactivity to both YSATLRY and YSPTLFY, and pAbs were purified using gels cross-linked with YSATLRYGGGSC. To confirm pAb specificity, a competition enzyme immunoassay was performed. The microtiter plate wells were coated with BSA-conjugated YSATLRYGGGSC and blocked with 3 % BSA in PBS. A pre-mixture of pAbs and peptides was added to each well. After washing with 0.05 % PBST three times, plates were incubated with HRP-conjugated anti-human IgG antibodies. Washing steps were repeated three times. ABTS in 0.05 M citric acid buffer (pH 4.0) and 1.0 % H_2_O_2_ were added to each well. OD was measured at 405 nm with a microplate spectrophotometer. LPCYTDHICYSSGGGS was used as a control peptide. (DOCX 75 kb) Additional file 5: Figure S3. Reactivity of scFv clones to peptides. (A) YSATLRY, YSPTRFY, and three CTD peptides (one unphosphorylated and two phosphorylated peptides) were synthesized and conjugated to BSA. (B) Microtiter plates were coated with BSA-conjugated peptide and blocked with 3 % BSA in PBS. Then, mAbs (as scFv-human Fc fusion proteins) diluted in 3 % BSA in PBS were added to individual wells. After washing with 0.05 % PBST three times, plates were incubated with HRP-conjugated anti-human Fc-specific antibodies. Washing steps were repeated three times. ABTS in 0.05 M citric acid buffer (pH 4.0) and 1.0 % H_2_O_2_ were added to each well. OD was measured at 405 nm with a microplate spectrophotometer. pAb 19 and normal human IgG were used as controls. (DOCX 168 kb) Additional file 6: Figure S4. Immunoprecipitation of LoVo cell lysates. LoVo cell lysates were incubated with either mAb 1 or mAb 2 (as scFv-human Fc fusion protein) conjugated to protein A beads. After washing, the immunoprecipitate was subjected to NuPage 4–12 % Bis-Tris gel electrophoresis (Lane 2). The gel was stained with Coomassie Brilliant Blue. Irrelevant scFv-human Fc fusion protein (irrelevant mAb) was used as a control in immunoprecipitation experiments. Lane 1 was loaded with the eluate of the mAb-conjugated gel without incubation with LoVo cell lysates. Bands 1 and 2 were expected to be RPB1 and RPB2 isoform 1, respectively. (DOCX 103 kb) Additional file 7: Figure S5. Correlation of ANA antibody index with antibody reactivity to YSATLRY and YSPTLFY. The sera of 45 centenarian, 25 old, and 25 young volunteers were tested for ANA using an enzyme immunoassay kit. Optical densities were converted to antibody index according to the manufacturer’s instructions. (DOCX 48 kb)
