# Supplementary material for: Antibodies to the DNA-directed RNA polymerase II subunit RPB1 occur with highest frequency in centenarians
Source: Immun Ageing. 2016 Mar 22;13:8. doi: 10.1186/s12979-016-0064-1 (PMC4802847; doi:10.1186/s12979-016-0064-1)
Supplement: Additional file 1: Table S1. — Age and gender distribution of subjects. (DOCX 18 kb) [file 12979_2016_64_MOESM1_ESM.docx]

**Additional files**

**Additional File 1**

**Additional file 1: Table S1.** Age and gender distribution of subjects

| Age group | Ages included | Gender | | | Age | |
| --- | --- | --- | --- | --- | --- | --- |
|  |  |  | N | % | Mean±SD | Range |
| Centenarians^[1]^ | ≥100 | M | 6 | 13.3 | 101.83±1.33 | 101-104 |
|  |  | F | 39 | 86.7 | 101.74±1.46 | 100-105 |
|  |  | Total | 45 | 100.0 | 101.76±1.43 | 100-105 |
| Old^[2]^ | 60~79 | M | 11 | 44 | 65.55±4.97 | 61-79 |
|  |  | F | 14 | 56 | 65.64±4.36 | 60-72 |
|  |  | Total | 25 | 100.0 | 65.60±4.54 | 60-79 |
| Young^[2]^ | ≤43 | M | 7 | 28 | 34.00±5.20 | 27-41 |
|  |  | F | 18 | 72 | 34.67±3.88 | 29-43 |
|  |  | Total | 25 | 100.0 | 34.48±4.18 | 27-43 |

^[1]^ J Gerontol a-Biol. 2003;58(3):227-31.

^[2]^ Hum Genet. 2006;119(3):353-4.
