# Supplementary material for: Antibodies to the DNA-directed RNA polymerase II subunit RPB1 occur with highest frequency in centenarians
Source: Immun Ageing. 2016 Mar 22;13:8. doi: 10.1186/s12979-016-0064-1 (PMC4802847; doi:10.1186/s12979-016-0064-1)
Supplement: Additional file 2: — Materials and Methods. (DOCX 29 kb) [file 12979_2016_64_MOESM2_ESM.docx]

**Additional File 2**

**Materials and Methods**

**Cell culture**

LoVo cells were obtained from the American Type Cell Collection (Manassas, VA, USA) and grown in RPMI-1640 media (Welgene, Seoul, South Korea) supplemented with 10% fetal bovine serum (GIBCO, Grand Island, NY, USA), 100 U/mL penicillin, and 100 μg/mL streptomycin at 37°C in a humidified atmosphere of 95% air and 5% CO_2_. HEK 293F cells (Invitrogen, Carlsbad, CA, USA) were grown in FreeStyle™ 293 Expression medium (GIBCO), containing 100 U/mL penicillin and 100 μg/mL streptomycin at 37°C in air containing 7% CO_2_ and 95% relative humidity (RH) on an orbital shaking incubator (Minitron, INFORS HT, Bottmingen, Switzerland) at 135 rpm.

**Preparation of human serum and antibodies**

Sera were collected from 45 centenarians [1] and 50 healthy volunteers [2]. The study was approved by the Institutional Review Board (IRB) of Samsung Medical Center (200412005). Briefly, 11, 22, and 12 centenarians were recruited from rural areas of Kyungsang, Chunra, and Cheju provinces in South Korea, respectively. A medical survey team visited the houses of centenarians and collected blood samples as a part of the survey procedure. The informed consent form was signed either by the subjects themselves or close relatives. To prepare polyclonal antibodies (pAbs) to YSATLRY, sera were collected from 59 additional healthy volunteers. The study was approved by the IRB of Seoul National University Hospital (C-0710-006-221). Written informed consent was obtained from all volunteers in accordance with the Declaration of Helsinki. Personal identifiers were removed, and data were analyzed anonymously.

**Biopanning for discovery of peptides reactive to the IgG fractions** **of** **centenarians**

We used three phage-displayed combinatorial peptide libraries: the random linear dodecapeptide library; the random linear heptapeptide library; and the random disulfide constrained heptapeptide library. All libraries were part of the Ph.D.^TM^ kit system (New England Biolabs, Ipswich, MA, USA).

IgG fractions were purified from human sera by affinity chromatography using a protein G Sepharose column (Santa Cruz Biotechnology, Santa Cruz, CA, USA) according to the manufacturer's instructions.

To enrich specific binding peptides from these combinatorial peptide libraries, a total of three rounds of biopanning were performed as described previously [3, 4]. Briefly, the IgG fractions were immobilized to protein G agarose beads, washed with phosphate-buffered saline (PBS), and blocked with 3% BSA in PBS (w/v) for 1 h at room temperature on a rotator. After blocking, beads were incubated with the phage-displayed combinatorial peptide library for 2 h at room temperature on a rotator and then washed with 0.05% Tween-20 in PBS (v/v, PBST) to remove unbound phage. The number of washes was increased to three times in subsequent rounds. After washing, 0.1 M glycine-HCl (pH 2.2) was added, and the beads were incubated for 10 min at room temperature to elute bound phage from the beads. Eluates were neutralized by adding 2 M Tris-Cl (pH 9.1). Then, the eluted phages were used to infect *E. coli* strain ER2738 cultures (New England Biolabs). After infection, cells were grown at 37°C overnight. Phages in the growth medium were precipitated by adding polyethylene glycol (PEG) 8000/NaCl. Two more cycles of biopanning were performed. In each round of biopanning, the eluted phages were titrated. Individual phage clones were isolated from the titration plate for output phages of the last round and infected using ER2738 cells. After overnight culture, phages that bound to centenarian IgG fractions were selected in phage enzyme immunoassays using centenarian IgG-coated microtiter plates as described previously [3]. Phage clones with positive signals were selected (OD_405_ ≥ 0.5), and their nucleotide sequences were determined as described previously [4].

**Phage enzyme immunoassay to test the reactivity of human sera to phage clones**

The selected phage clones that recognized YSATLRY or YSPTLFY were used to infect exponentially growing ER2738 cells. After overnight growth at 37°C, the culture supernatant was used to perform the phage enzyme immunoassay as described previously [3]. Microtiter plates (Corning Costar Corp., Cambridge, MA, USA) were coated with anti-human IgG (Fab specific) antibodies (Sigma, St. Louis, MO, USA), incubated overnight at 4°C, and blocked with 3% BSA in PBS. Individual human serum diluted in 3% BSA in PBS was added to individual wells, and the plates were incubated for 2 h at 37°C. After washing with 0.05% PBST three times, phages displaying either YSATLRY or YSPTLFY in the culture supernatant were mixed with an equal volume of 6% BSA in PBS and incubated for 2 h at 37°C. After washing with 0.05% PBST three times, the plates were incubated with HRP-conjugated anti-M13 antibodies (GE Healthcare Life Sciences, Piscataway, NJ, USA). Plates were washed with 0.05% PBST five times, and then 2,2′-azino-bis(3-ethylbenzothiazoline-6-sulphonic acid) (ABTS, Amresco, Solon, OH, USA) in 0.05 M citric acid buffer (pH 4.0) and 1.0 % H_2_O_2_ were added to each well. After incubation for 30 min at 37°C, optical density (OD) was measured at 405 nm with a microplate spectrophotometer (Labsystems Multiskan, Thermo Fisher Scientific, Rockford, IL, USA).

**Peptide synthesis and conjugation**

All peptides used in this study were synthesized by Peptron, Inc. (Daejeon, South Korea). YSATLRYGGGSC, YSPTLFYGGGSC, irrelevant control peptide LPCYTDHICYSSGGGSC, CTD (YSPTSPSYSPTSPSC), pS5S2 (YSPTpSPSYpSPTSPSC), and pS5 (YSPTpSPSYSPTSPSC) were synthesized and conjugated to BSA as described previously [4].

**Enzyme immunoassay**

Microtiter plates were coated with BSA-conjugated YSATLRYGGGSC and YSAPLFYGGGSC peptides overnight at 4°C and blocked with 3% BSA in PBS. Individual human serum was diluted with 3% BSA in PBS and incubated for 2 h at 37°C. After washing with 0.05% PBST three times, plates were incubated with HRP-conjugated anti-human IgG (H+L) antibodies (Jackson ImmunoResearch Inc., West Grove, PA, USA). Plates were washed with 0.05% PBST three times. ABTS in 0.05 M citric acid buffer (pH 4.0) and 1.0 % H_2_O_2_ were added to each well. OD was measured at 405 nm with a microplate spectrophotometer. BSA-conjugated YSATLRYGGGSC, YSAPLFYGGGSC, YSPTSPSYSPTSPSC, YSPTpSPSYpSPTSPSC, and YSPTpSPSYSPTSPSC peptides and mAb 1, mAb 2, and pAb 19 were used in Additional Figure 3 following the same procedures.

**Purification of pAbs from human serum**

A YSATLRYGGGSC-cross-linked affinity column was prepared using a Sulfolink kit (Pierce Biotechnology, Inc., Rockford, IL, USA), and pAbs were purified from five volunteers’ sera (Volunteer #7, #11, #19, #47, and #50) according to the manufacturer’s instructions.

**Immunoprecipitation, gel electrophoresis, and protein staining**

LoVo cells were lysed with lysis buffer [20 mM Tris-HCl (pH 7.5), 150 mM NaCl, 1% (v/v) Triton X-100, 0.25% (v/v) Na-deoxycholate, 1 mM EDTA, 1× protease inhibitor cocktail (Amresco), and 1× phosphatase inhibitor cocktail (Roche, Basel, Switzerland)], and incubated with anti-YSATLRY pAbs (pAb 47, pAb 19, pAb 11, pAb 50, and pAb 7) or mAbs (mAb 1 and mAb 2) overnight at 4°C. Then, protein A agarose beads (Repligen, Waltham, MA, USA) were added and incubated for 2 h at 4°C. The beads were washed four times with lysis buffer. SDS sample buffer was added to the beads. The beads were boiled for 5 min and centrifuged at 5,000 rpm, and the supernatant fractions were loaded onto a NuPage 4−12% Bis-Tris gel (Invitrogen). Gel electrophoresis was performed following the manufacturer's instructions. The gel was stained with Coomassie Brilliant Blue R-250 (Amresco) to visualize protein bands.

**Mass spectrometry analysis**

Mass spectrometry was performed by the Peptide Library Support Facility, POSTECH (Pohang, South Korea) on request. Samples were analyzed using the Applied Biosystems 4700 proteomics analyzer (SCIEX, Forster City, CA, USA). Protein identification using peptide sequences from LC–MS/MS samples was performed using MASCOT software as described previously [5].

**Immunoblot analysis**

Immunoprecipitates or whole cell lysates were subjected to electrophoresis on a NuPage 4−12% Bis-Tris gel, and resolved proteins were transferred to a nitrocellulose membrane (Whatman, Kent, UK) as described previously [6]. The membrane was blocked with 5% non-fat dry milk (BD Biosciences, Sparks, MD, USA) with 0.1% Tween-20 in TBS (v/v, TBST) at room temperature for 1 h. The membrane was then incubated with primary antibodies (anti-pan CTD, phosphorylated serine 2 CTD, and phosphorylated serine 5 CTD were from Abcam, Cambridge Science Park, Cambridge, UK) diluted in 5% non-fat dry milk in 0.1% TBST overnight at 4°C. After washing four times with 0.1% TBST, the membrane was incubated with HRP-conjugated secondary antibodies (anti-mouse IgG Fc-specific HRP was from Sigma; anti-rabbit IgG Fc-specific HRP and anti-rat IgG H+L-specific HRP were from Jackson ImmunoResearch Inc.) diluted in 5% non-fat dry milk in 0.1% TBST for 1 h at room temperature. The membrane was washed four times with 0.1% TBST, and proteins were visualized by SuperSignal Pico West chemiluminescent substrate (Thermo Fisher Scientific).

**Human antibody library construction and biopanning**

Peripheral mononuclear cell fractions were isolated from the blood of volunteer #19 using Ficoll-Paque PLUS (GE Healthcare Life Sciences) following the manufacturer’s instructions. The phage-displayed combinatorial single-chain variable fragment (scFv) library was constructed using total RNA isolated from peripheral mononuclear cell fractions of volunteer #19 as described previously [3].

To enrich specific phages from the library, a total of four rounds of biopanning were performed as described previously [3]. First, 3 μg of YSATLRYGGGSC-BSA conjugate was conjugated to 5.0 × 10^6^ paramagnetic beads (M-270 Epoxy DynaBeads, Invitrogen) following the manufacturer’s instructions and blocked with 3% BSA in PBS. After blocking, beads were incubated with the phage-displayed combinatorial scFv library for 2 h at room temperature on a rotator and washed with 0.05% PBST to remove unbound phage; the number of washes was increased to three times in subsequent rounds. After washing, 0.1 M glycine-HCl (pH 2.2) was added to the beads and incubated for 10 min at room temperature to elute bound phage from the beads. The eluate was neutralized by the addition of 2 M Tris-Cl (pH 9.1). Then, the eluted phages were used to infect *E. coli* strain ER2738 cultures. After infection, cells were grown at 37°C overnight, and the scFv-displaying phages were rescued by the addition of helper phages. The phages present in the growth medium were precipitated by adding PEG 8000/NaCl as described previously [3]. Three more cycles of biopanning were performed. In each round of biopanning, the eluted phages were titrated. Individual phage clones were isolated from the titration plate for output phages of the last round and used to infect ER2738 cells.

After overnight culture, the culture supernatant was subjected to a phage enzyme immunoassay using microtiter plates coated with YSATLRY and YSPTLFY peptides as described previously [3]. The phage clones with positive signals were selected (OD_405_ ≥ 0.5), and their nucleotide sequences were determined as described previously [4].

**Expression and purification of scFv-Fc fusion protein**

An expression vector modified from pCEP4 (Invitrogen) was employed [7]. The vector has an expression cassette containing the leader sequence of the human Ig κ-chain, two *sfi*I sites for insertion of the antibody gene of interest, hinge region of human IgG_1_, and the C_H2_-C_H3_ domains of human IgG.

The phagemid DNA of selected clones and the expression vector were digested with *sfi*I, and the scFv genes were cloned into the expression vector. The expression vector was then transfected into HEK 293F cells using 25 kDa linear polyethylenimine (Polysciences, Warrington, PA, USA) as described previously [8]. The scFv-Fc fusion proteins in the culture supernatant were purified by affinity chromatography using a protein A Sepharose column according to the manufacturer’s instructions.

**Determination of antinuclear antibody (ANA) IgG level in the sera**

The level of ANA IgG in the sera was determined using a commercially available enzyme immunoassay kit (Abnova, Taipei, Taiwan) following the manufacturer’s instructions. Briefly, sera were diluted 1:21 with the sample diluent included in the kit. Then, 100 μl each of the diluted sera, calibrator, and controls were added into separate wells and incubated at room temperature for 20 min. After washing, enzyme conjugate was added into each well. The plate was then incubated at room temperature for 20 min before washing. TMB substrate solution was added into each well and the plate was incubated at room temperature for 10 min. After the stop solution was added, the OD was measured at 450 nm with a microplate spectrophotometer (Labsystems Multiskan, Thermo Fisher Scientific). OD values were converted to antibody indices according to the manufacturer’s instructions. When the antibody index was higher than 1.1, the sample was considered to have a detectable level of ANA IgG.

**Additional References**

1. Choi YH, Kim JH, Kim DK, Kim JW, Kim DK, Lee MS et al. Distributions of ACE and APOE polymorphisms and their realations with dementia status in Korean centenarians. J Gerontol a-Biol. 2003;58(3):227-31.

2. Kim DJ, Yi SM, Lee SY, Kang HS, Choi YH, Song YW et al. Association between the MLH1 gene and lonevity. Hum Genet. 2006;119(3):353-4.

3. Barbas CF. Phage display : a laboratory manual. Cold Spring Harbor, NY: Cold Spring Harbor Laboratory Press; 2001.

4. Chung J, Park S, Kim D, Rhim J, Kim I, Choi I et al. Identification of antigenic peptide recognized by the anti-JL1 leukemia-specific monoclonal antibody from combinatorial peptide phage display libraries. J Cancer Res Clin. 2002;128(12):641-9.

5. Perkins DN, Pappin DJC, Creasy DM, Cottrell JS. Probability-based protein identification by searching sequence databases using mass spectrometry data. Electrophoresis. 1999;20(18):3551-67.

6. Burnette WN. Western Blotting - Electrophoretic Transfer of Proteins from Sodium Dodecyl Sulfate-Polyacrylamide Gels to Unmodified Nitrocellulose and Radiographic Detection with Antibody and Radioiodinated Protein-A. Anal Biochem. 1981;112(2):195-203.

7. Park S, Lee DH, Park JG, Lee YT, Chung J. A sensitive enzyme immunoassay for measuring cotinine in passive smokers. Clinica Chimica Acta. 2010;411(17-18):1238-42.

8. Boussif O, Lezoualch F, Zanta MA, Mergny MD, Scherman D, Demeneix B et al. A Versatile Vector for Gene and Oligonucleotide Transfer into Cells in Culture and in-Vivo - Polyethylenimine. P Natl Acad Sci USA. 1995;92(16):7297-301.
