# Supplementary material for: Antibodies to the DNA-directed RNA polymerase II subunit RPB1 occur with highest frequency in centenarians
Source: Immun Ageing. 2016 Mar 22;13:8. doi: 10.1186/s12979-016-0064-1 (PMC4802847; doi:10.1186/s12979-016-0064-1)
Supplement: Additional file 3: Figure S1. — Enzyme immunoassay to determine antibody titer to YSATLRY (A) and YSPTLFY (B). Wells of a microtiter plate were coated with either YSATLRYGGGSC or YSPTLFYGGGSC peptide conjugated to BSA and blocked with 3 % BSA in PBS. Sera from individuals diluted in 3 % BSA in PBS were added to the wells. After washing with 0.05 % PBST three times, the plates were incubated with HRP-conjugated anti-human IgG antibodies. The washing steps were repeated three times. 2,2′-azino-bis(3-ethylbenzothiazoline-6-sulphonic acid (ABTS) in 0.05 M citric acid buffer (pH 4.0) and 1.0 % H2O2 were added to each well. OD was measured at 405 nm with a microplate spectrophotometer. Asterisks indicate five selected volunteers for further studies (Volunteer #7 = pAb 7, Volunteer #11 = pAb 11, Volunteer #19 = pAb 19, Volunteer #47 = pAb 47, and Volunteer #50 = pAb 50). (DOCX 150 kb) [file 12979_2016_64_MOESM3_ESM.docx]

**Additional File 3**


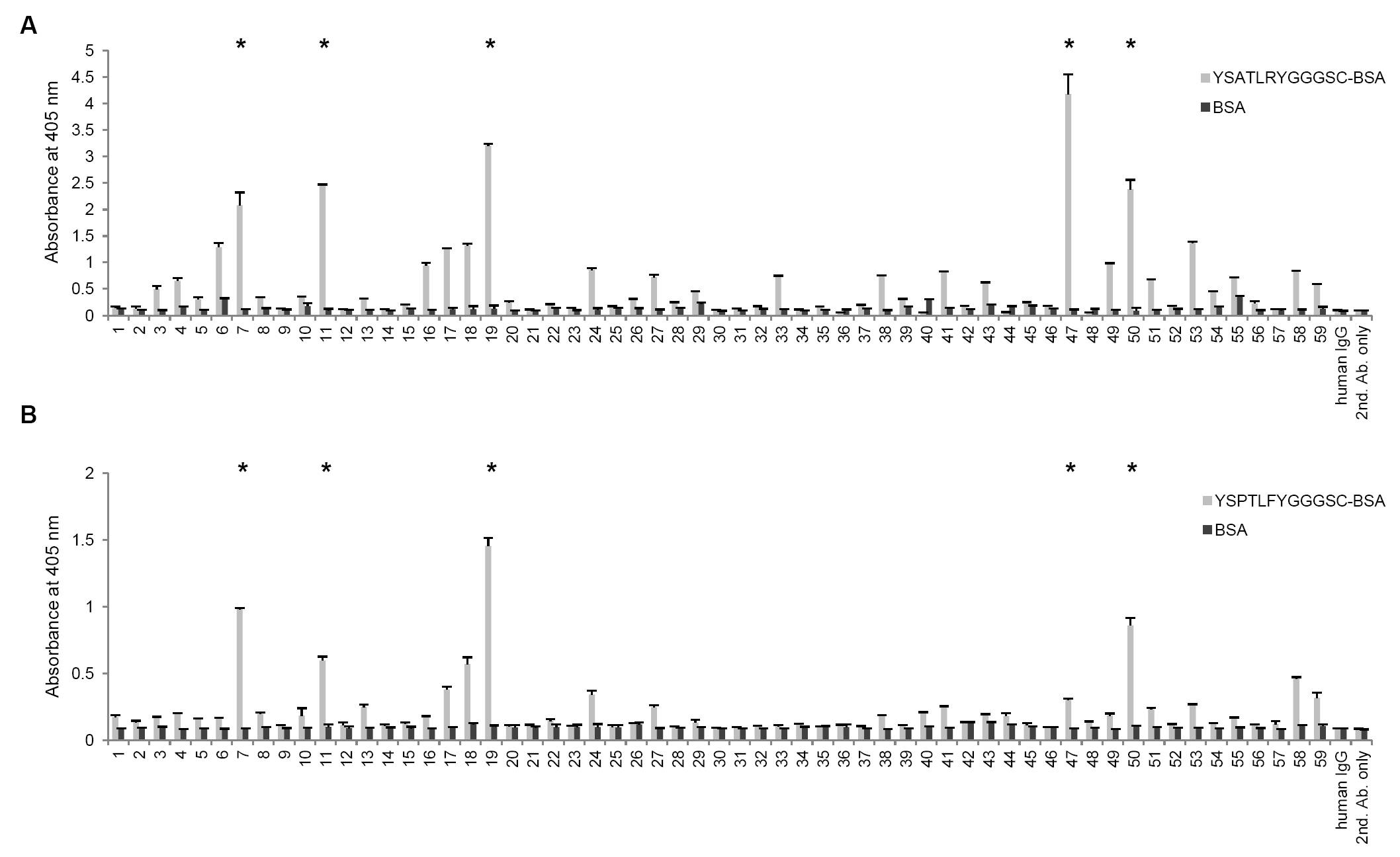


**Additional file 3: Fig. S1.** Enzyme immunoassay to determine antibody titer to YSATLRY (A) and YSPTLFY (B). Wells of a microtiter plate were coated with either YSATLRYGGGSC or YSPTLFYGGGSC peptide conjugated to BSA and blocked with 3% BSA in PBS. Sera from individuals diluted in 3% BSA in PBS were added to the wells. After washing with 0.05% PBST three times, the plates were incubated with HRP-conjugated anti-human IgG antibodies. The washing steps were repeated three times. 2,2′-azino-bis(3-ethylbenzothiazoline-6-sulphonic acid (ABTS) in 0.05 M citric acid buffer (pH 4.0) and 1.0 % H_2_O_2_ were added to each well. OD was measured at 405 nm with a microplate spectrophotometer. Asterisks indicate five selected volunteers for further studies (Volunteer #7 = pAb 7, Volunteer #11 = pAb 11, Volunteer #19 = pAb 19, Volunteer #47 = pAb 47, and Volunteer #50 = pAb 50).
