# Supplementary material for: Antibodies to the DNA-directed RNA polymerase II subunit RPB1 occur with highest frequency in centenarians
Source: Immun Ageing. 2016 Mar 22;13:8. doi: 10.1186/s12979-016-0064-1 (PMC4802847; doi:10.1186/s12979-016-0064-1)
Supplement: Additional file 4: Figure S2. — Competition enzyme immunoassay using polyclonal antibodies (pAbs) with the peptide YSATLRY. Sera from five volunteers showed reactivity to both YSATLRY and YSPTLFY, and pAbs were purified using gels cross-linked with YSATLRYGGGSC. To confirm pAb specificity, a competition enzyme immunoassay was performed. The microtiter plate wells were coated with BSA-conjugated YSATLRYGGGSC and blocked with 3 % BSA in PBS. A pre-mixture of pAbs and peptides was added to each well. After washing with 0.05 % PBST three times, plates were incubated with HRP-conjugated anti-human IgG antibodies. Washing steps were repeated three times. ABTS in 0.05 M citric acid buffer (pH 4.0) and 1.0 % H2O2 were added to each well. OD was measured at 405 nm with a microplate spectrophotometer. LPCYTDHICYSSGGGS was used as a control peptide. (DOCX 75 kb) [file 12979_2016_64_MOESM4_ESM.docx]

**Additional File 4**


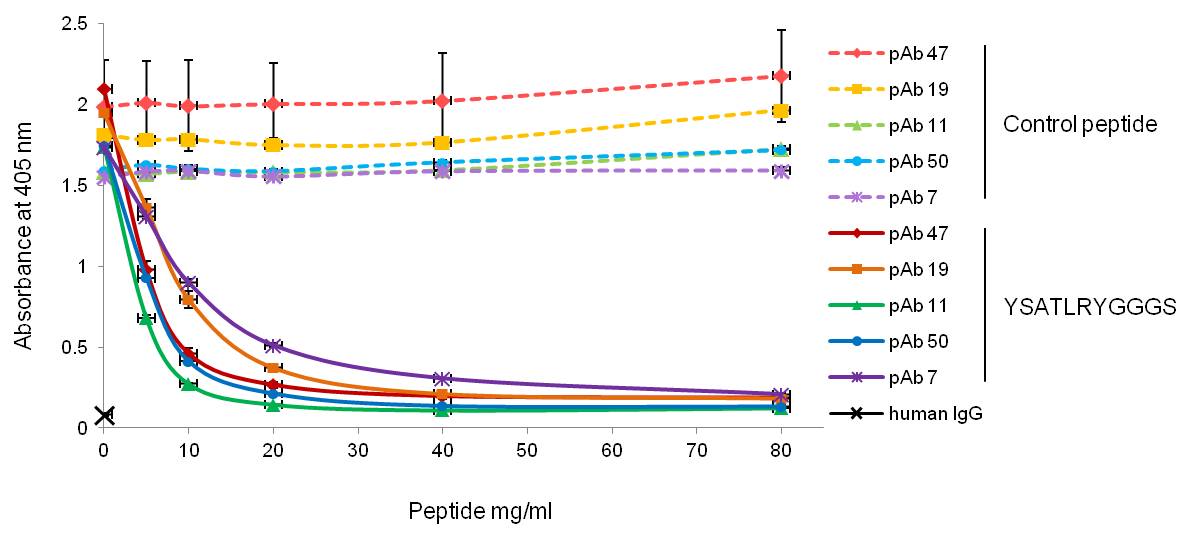


**Additional file 4: Fig. S2.** Competition enzyme immunoassay using polyclonal antibodies (pAbs) with the peptide YSATLRY. Sera from five volunteers showed reactivity to both YSATLRY and YSPTLFY, and pAbs were purified using gels cross-linked with YSATLRYGGGSC. To confirm pAb specificity, a competition enzyme immunoassay was performed. The microtiter plate wells were coated with BSA-conjugated YSATLRYGGGSC and blocked with 3% BSA in PBS. A pre-mixture of pAbs and peptides was added to each well. After washing with 0.05% PBST three times, plates were incubated with HRP-conjugated anti-human IgG antibodies. Washing steps were repeated three times. ABTS in 0.05 M citric acid buffer (pH 4.0) and 1.0 % H_2_O_2_ were added to each well. OD was measured at 405 nm with a microplate spectrophotometer. LPCYTDHICYSSGGGS was used as a control peptide.
