# Supplementary material for: Antibodies to the DNA-directed RNA polymerase II subunit RPB1 occur with highest frequency in centenarians
Source: Immun Ageing. 2016 Mar 22;13:8. doi: 10.1186/s12979-016-0064-1 (PMC4802847; doi:10.1186/s12979-016-0064-1)
Supplement: Additional file 5: Figure S3. — Reactivity of scFv clones to peptides. (A) YSATLRY, YSPTRFY, and three CTD peptides (one unphosphorylated and two phosphorylated peptides) were synthesized and conjugated to BSA. (B) Microtiter plates were coated with BSA-conjugated peptide and blocked with 3 % BSA in PBS. Then, mAbs (as scFv-human Fc fusion proteins) diluted in 3 % BSA in PBS were added to individual wells. After washing with 0.05 % PBST three times, plates were incubated with HRP-conjugated anti-human Fc-specific antibodies. Washing steps were repeated three times. ABTS in 0.05 M citric acid buffer (pH 4.0) and 1.0 % H2O2 were added to each well. OD was measured at 405 nm with a microplate spectrophotometer. pAb 19 and normal human IgG were used as controls. (DOCX 168 kb) [file 12979_2016_64_MOESM5_ESM.docx]

**Additional File 5**


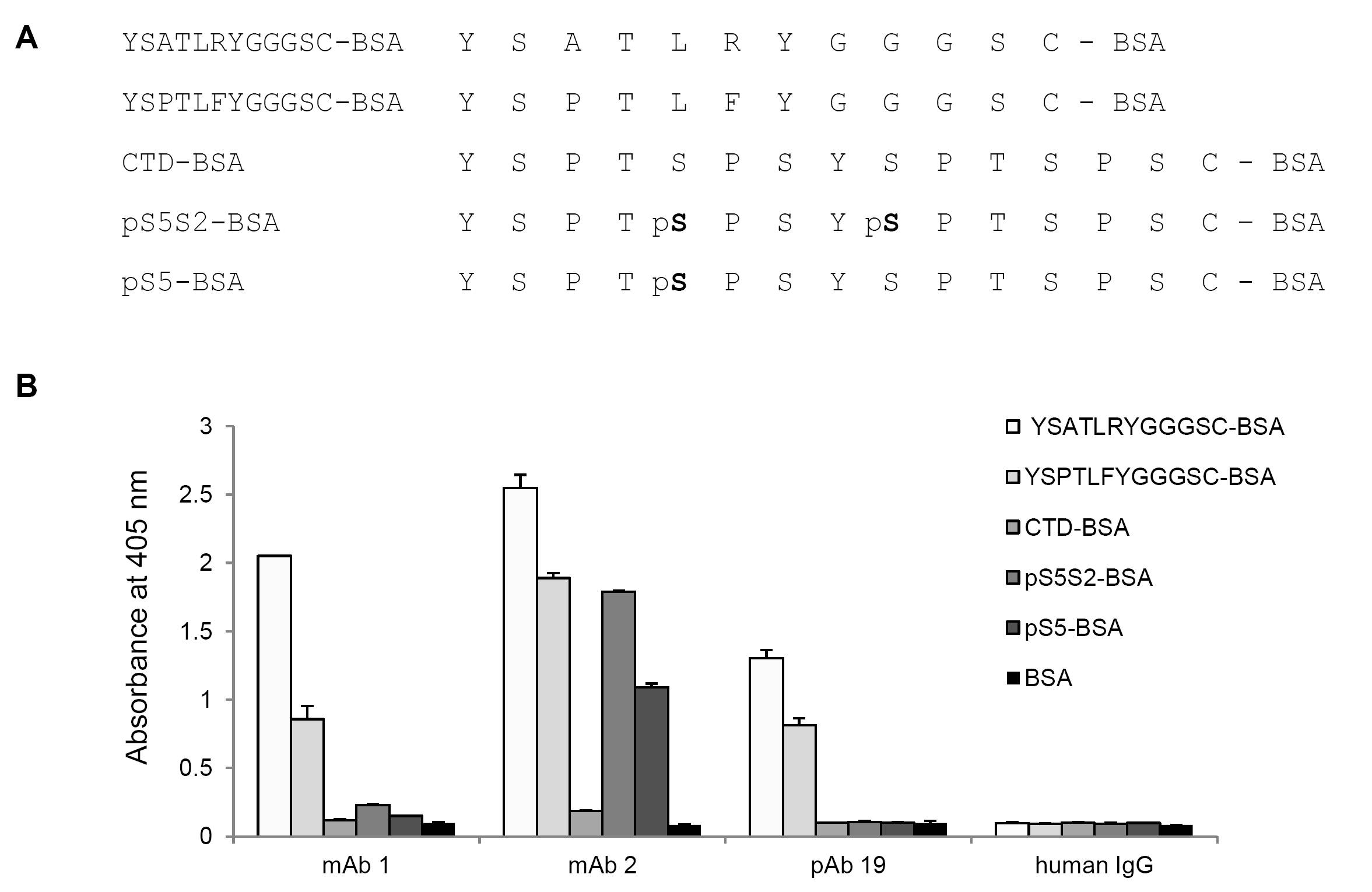


**Additional file 5: Fig. S3.** Reactivity of scFv clones to peptides. (A) YSATLRY, YSPTRFY, and three CTD peptides (one unphosphorylated and two phosphorylated peptides) were synthesized and conjugated to BSA. (B) Microtiter plates were coated with BSA-conjugated peptide and blocked with 3% BSA in PBS. Then, mAbs (as scFv-human Fc fusion proteins) diluted in 3% BSA in PBS were added to individual wells. After washing with 0.05% PBST three times, plates were incubated with HRP-conjugated anti-human Fc-specific antibodies. Washing steps were repeated three times. ABTS in 0.05 M citric acid buffer (pH 4.0) and 1.0 % H_2_O_2_ were added to each well. OD was measured at 405 nm with a microplate spectrophotometer. pAb 19 and normal human IgG were used as controls.
