# Supplementary material for: Antibodies to the DNA-directed RNA polymerase II subunit RPB1 occur with highest frequency in centenarians
Source: Immun Ageing. 2016 Mar 22;13:8. doi: 10.1186/s12979-016-0064-1 (PMC4802847; doi:10.1186/s12979-016-0064-1)
Supplement: Additional file 6: Figure S4. — Immunoprecipitation of LoVo cell lysates. LoVo cell lysates were incubated with either mAb 1 or mAb 2 (as scFv-human Fc fusion protein) conjugated to protein A beads. After washing, the immunoprecipitate was subjected to NuPage 4–12 % Bis-Tris gel electrophoresis (Lane 2). The gel was stained with Coomassie Brilliant Blue. Irrelevant scFv-human Fc fusion protein (irrelevant mAb) was used as a control in immunoprecipitation experiments. Lane 1 was loaded with the eluate of the mAb-conjugated gel without incubation with LoVo cell lysates. Bands 1 and 2 were expected to be RPB1 and RPB2 isoform 1, respectively. (DOCX 103 kb) [file 12979_2016_64_MOESM6_ESM.docx]

**Additional File 6**


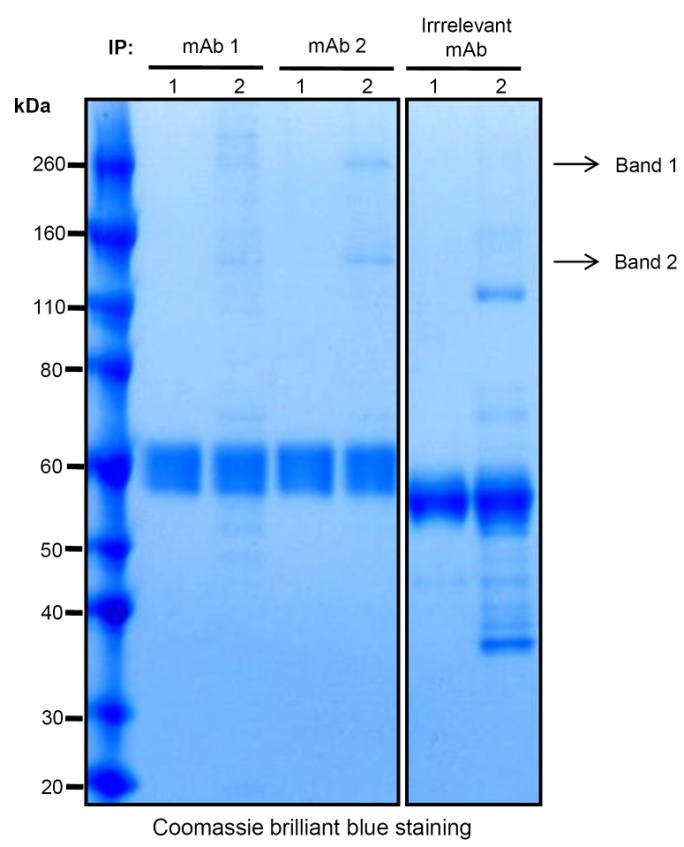


**Additional file 6: Fig. S4.** Immunoprecipitation of LoVo cell lysates. LoVo cell lysates were incubated with either mAb 1 or mAb 2 (as scFv-human Fc fusion protein) conjugated to protein A beads. After washing, the immunoprecipitate was subjected to NuPage 4−12% Bis-Tris gel electrophoresis (Lane 2). The gel was stained with Coomassie Brilliant Blue. Irrelevant scFv-human Fc fusion protein (irrelevant mAb) was used as a control in immunoprecipitation experiments. Lane 1 was loaded with the eluate of the mAb-conjugated gel without incubation with LoVo cell lysates. Bands 1 and 2 were expected to be RPB1 and RPB2 isoform 1, respectively.
