# Supplementary material for: Antibodies to the DNA-directed RNA polymerase II subunit RPB1 occur with highest frequency in centenarians
Source: Immun Ageing. 2016 Mar 22;13:8. doi: 10.1186/s12979-016-0064-1 (PMC4802847; doi:10.1186/s12979-016-0064-1)
Supplement: Additional file 7: Figure S5. — Correlation of ANA antibody index with antibody reactivity to YSATLRY and YSPTLFY. The sera of 45 centenarian, 25 old, and 25 young volunteers were tested for ANA using an enzyme immunoassay kit. Optical densities were converted to antibody index according to the manufacturer’s instructions. (DOCX 48 kb) [file 12979_2016_64_MOESM7_ESM.docx]

**Additional File 7**


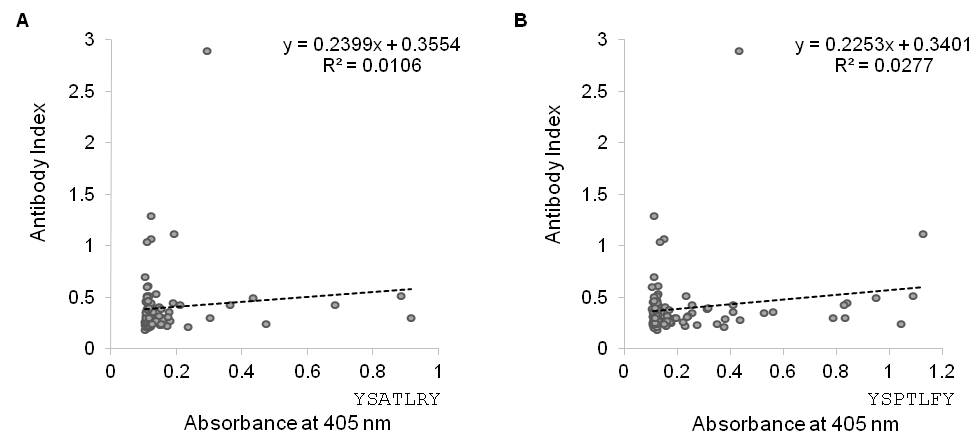


**Additional file 7: Fig. S5.** Correlation of ANA antibody index with antibody reactivity to YSATLRY and YSPTLFY. The sera of 45 centenarian, 25 old, and 25 young volunteers were tested for ANA using an enzyme immunoassay kit. Optical densities were converted to antibody index according to the manufacturer’s instructions.
